# Starch Trek: The Search for Yield

**DOI:** 10.3389/fpls.2018.01930

**Published:** 2019-01-21

**Authors:** James R. Lloyd, Jens Kossmann

**Affiliations:** Department of Genetics, Institute for Plant Biotechnology, University of Stellenbosch, Stellenbosch, South Africa

**Keywords:** starch, sucrose, carbohydrate partitioning, photosynthesis, metabolism

## Abstract

Starch is a plant storage polyglucan that accumulates in plastids. It is composed of two polymers, amylose and amylopectin, with different structures and plays several roles in helping to determine plant yield. In leaves, it acts as a buffer for night time carbon starvation. Genetically altered plants that cannot synthesize or degrade starch efficiently often grow poorly. There have been a number of successful approaches to manipulate leaf starch metabolism that has resulted in increased growth and yield. Its degradation is also a source of sugars that can help alleviate abiotic stress. In edible parts of plants, starch often makes up the majority of the dry weight constituting much of the calorific value of food and feed. Increasing starch in these organs can increase this as well as increasing yield. Enzymes involved in starch metabolism are well known, and there has been much research analyzing their functions in starch synthesis and degradation, as well as genetic and posttranslational regulatory mechanisms affecting them. In this mini review, we examine work on this topic and discuss future directions that could be used to manipulate this metabolite for improved yield.

## Introduction

The need for improved crop yields due to an increase in world population and a decrease in available agricultural land is well known (Edgerton, 2009). This problem will likely be exacerbated through alterations in environmental conditions caused by anthropomorphic CO_2_ release that may lead to increases in both biotic and abiotic stresses (Fodor et al., 2017). There are many potential biotechnological methods that can lead to increased yield, and one of these involves altering starch metabolism. This mini review will examine work that has been performed to improve plant yield through manipulation of this metabolite and suggest new avenues that could be explored.

Starch is a polyglucan that is stored as granules within plastids. It consists of two polymers with differing structures, amylose and amylopectin. Amylose contains relatively long (normally composed of several hundred glucose monomers) α1,4 linked chains, while amylopectin is composed of many short (approximately 5–50 glucose monomers) α1,4 linked chains, linked together by α1,6 branch points in an ordered, crystalline array (Zeeman et al., 2010).

Manipulation of starch metabolism is important for improving plant yield for several reasons. As starch is the major form of calories within plants, increasing starch concentrations in plant tissues can mean that less food or fodder has to be consumed to supply the same energetic value (Ruckle et al., 2017). It is also a major sink within storage organs, so increasing starch here can lead to increased plant yield simply as more accumulates. In leaves, it plays two roles. Firstly, it is synthesized during the day and degraded at night, buffering the plant from night time carbon starvation (Stitt and Zeeman, 2012; Arias et al., 2014) which leads to transcriptional upregulation of stress-related genes (Stitt et al., 2007) and an inhibition of gibberellin synthesis (Paparelli et al., 2013). Secondly, it is a source of carbon skeletons for the production of compatible solutes that help plants to overcome abiotic stress (Thalmann and Santelia, 2017). Finally, starch has been shown to affect developmental processes (Matsoukas et al., 2013), and therefore, its manipulation could increase yield through altering plant or seed development.

### Starch Metabolism

Due to its many roles, the pathway of starch metabolism has been studied intensively, and many enzymatic steps involved in its metabolism have been elucidated. This knowledge has been used to construct a detailed model of its metabolism, which has been described in several recent reviews (Bahaji et al., 2014; Pfister and Zeeman, 2016; MacNeill et al., 2017). The rest of this section will briefly outline the major enzymes involved to allow for further discussion of the manipulation of the pathway.

**Figure 1:**
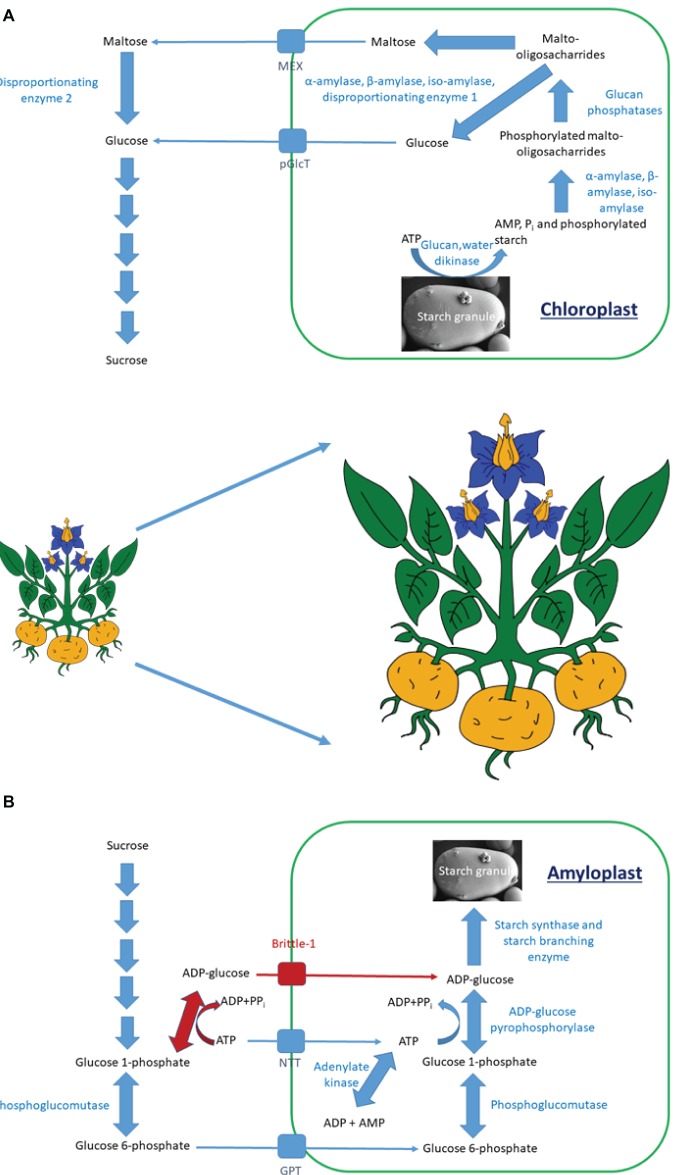
The pathway of starch degradation in **(A)** leaves and of starch synthesis in **(B)** storage organs. **(A)** Starch is degraded through a series of enzymatic steps to maltose and glucose. These are transported from the chloroplast by either the maltose export (MEX) or plastidial glucose transport (pGlcT) proteins. Within the cytosol, they are converted to sucrose for further transport. **(B)** There are two pathways of starch synthesis in vascular plants. One is shared in all plants (blue arrows), while the other is present only in cereal endosperm (red arrows). Sucrose is degraded to glucose 6-phosphate, which is transported into amyloplasts by the glucose 6-phosphate/phosphate transporter (GPT). The ADP-glucose pyrophosphorylase step within the amyloplast utilizes ATP, which is imported into the amyloplast by the plastidial ATP/ADP transporter (NTT). Within cereal endosperm, ADP-glucose is synthesized extraplastidially and is imported into amyloplasts by the Brittle-1 transporter.

Starch polymer formation (Figure 1B) involves the synthesis of adenosine diphosphate (ADP)-glucose by ADP-glucose pyrophosphorylase (AGPase). This is used by starch synthases (SS) to form linear α1,4 glucan chains. One starch synthase isoform is responsible for amylose synthesis, while several others are involved in granule initiation and amylopectin synthesis (Pfister and Zeeman, 2016; Nazarian-Firouzabadi and Visser, 2017). The branch points within amylopectin are introduced by starch branching enzyme (SBE) isoforms, while excess α1,6 links are removed by isoamylases (Pfister and Zeeman, 2016; MacNeill et al., 2017).

Starch degradation (Figure 1A) is initiated by glucan water dikinase (GWD) enzymes that phosphorylate the granule, helping to solubilize it and allow access to α-, β-, and isoamylases (Streb et al., 2012; Mahlow et al., 2016). These release phosphorylated malto-oligosaccharides into the stroma, where the phosphate is removed by glucan phosphatases, allowing further degradation to maltose and glucose by the combined actions of amylases and disproportionating enzyme 1. Maltose and glucose are exported to the cytosol through specific transport proteins, where maltose becomes further mobilized by disproportionating enzyme 2 (DPE2; Lloyd and Kossmann, 2015).

The regulation of starch metabolism takes place at several levels, including both transcriptional and posttranslational mechanisms. A number of transcription factors affecting expression of genes encoding starch metabolizing enzymes have been identified, and their roles are currently being examined. At the posttranslational level, allosteric regulation (Zeeman et al., 2010) protein phosphorylation (Kötting et al., 2010) and reducing/oxidation (redox) conditions (Santelia et al., 2015; Skryhan et al., 2018) are known to influence both enzyme activities and the formation of enzyme complexes. The roles of these processes will probably differ between leaves and heterotrophic storage organs. For example, the redox status within chloroplasts varies over a day/night cycle, where reducing condition predominates during the day and oxidizing conditions at night. Within amyloplasts of heterotrophic tissues, this day/night cycle will not occur, although it has been shown that the alteration in redox status of chloroplasts can be sensed by amyloplasts (Balmer et al., 2006). Finally, nutrient sensing mechanisms would be expected to link the accumulation of soluble sugars, such as sucrose, with starch synthesis through a combination of mechanisms including 14-3-3 proteins, hexokinase (Rolland et al., 2006), SnRKs (Crozet et al., 2014; Wurzinger et al., 2018), and TOR kinases (Rolland et al., 2006; Dobrenel et al., 2016; Shi et al., 2018).

### ADP-Glucose Pyrophosphorylase—A Key Enzyme for Yield in Both Storage Organs and Leaves

ADP-glucose pyrophosphorylase (Figure 1B) has been shown to be a key enzyme influencing starch accumulation in both leaves and storage organs. One complicating factor is the level of posttranslational control of the enzyme by a combination of allosteric effectors and redox, which means that overexpression of the native enzyme may not increase flux into starch synthesis (Boehlein et al., 2013a). This has been overcome through the use of mutated sequences encoding allosteric and redox insensitive isoforms (Tuncel and Okita, 2013). In cereal endosperm, another complication is that most AGPase activity is found within the cytosol, with a specific transporter localized in the outer plastid membrane importing ADP-glucose into the stroma, while in noncereal species it is found only in the plastid. Within cereal endosperm, the cytosolic pathway plays a greater role than the plastidial one (Tuncel and Okita, 2013; Tetlow and Emes, 2017).

It has been known for many years that increasing AGPase activity leads to increased starch synthesis, and as was mentioned above, increasing starch in food and fodder crops would improve their calorific value (Ruckle et al., 2017, 2018). However, interestingly, increasing starch amounts in this way can have beneficial effects on plant productivity. In Arabidopsis and rice, where leaf AGPase activity has been upregulated, leaf starch amounts are increased at the end of the day and the plants grow larger (Gibson et al., 2011; Schlosser et al., 2014; Oiestad et al., 2016), most likely caused by elevated nighttime sugar levels (Stitt and Zeeman, 2012; Arias et al., 2014).

Increasing AGPase in storage organs can also increase starch amounts (Zeeman et al., 2010; Sonnewald and Kossmann, 2013; Tuncel and Okita, 2013). Initial work utilized a bacterial gene that was insensitive to allosteric regulation (Stark et al., 1992); however, more recent work has used plant genes engineered to encode proteins with improved properties (Tuncel and Okita, 2013). Due to the dual cytosolic and plastidial localization of AGPase in cereal endosperm (Figure 1B) compared to its plastidial targeting in other organs and species, the subcellular targeting of AGPase is critical for successful upregulation of starch synthesis. In noncereal species, transgenic plants with increased plastidial AGPase activity accumulate increased amounts of starch, while in cereal endosperm the enzyme has to be localized to the cytosol to have an effect (Zeeman et al., 2010; Sonnewald and Kossmann, 2013; Tuncel and Okita, 2013).

It is clear that AGPase plays a critical role in determining starch yield. It is important to understand, therefore, how its properties could be altered to increase yield, especially in a changing environment. Protein engineering using plant genes has led to much knowledge about reducing redox or allosteric inhibition of its activity as well as improving its activity under heat stress (Georgelis and Hannah, 2008; Haedrich et al., 2012; Boehlein et al., 2013a,b, 2015), and many of these engineered proteins have been transferred into plants through transgenic technology (Tuncel and Okita, 2013). A recent TILLING population targeting this enzyme in Arabidopsis has been developed and may help in developing nontransgenic routes to increase starch synthesis *in vivo* using gene editing (Haedrich et al., 2011). Although increasing AGPase activity will increase flux into the pathway, this is not the only way that it increases yield. In several cereals, for example, over-expression of AGPase can increase seed number, which in maize has been shown to occur through an effect on maternal tissues (Smidansky et al., 2002, 2003; Hannah et al., 2012, 2017).

Another strategy used to influence the AGPase step in storage organs has been to increase supply of its substrates, adenosine triphosphate (ATP) and/or glucose 6-phosphate. In dicots, ATP limitation of AGPase within plastids has been suggested (Geigenberger, 2001), meaning that increasing supply would be a necessity to increase starch contents. This has been achieved in potato through overexpression of a plastidial adenylate translocator (NTT; which counter-exchanges ATP for ADP; Figure 1B). However, reports on the effect of increasing *NTT* expression on starch amounts are mixed. Two studies in potato found that this increased starch amounts (Tjaden et al., 1998; Geigenberger, 2001) while another described no alteration (Zhang et al., 2008). That later study did, however, find that combined overexpression of the NTT and a plastidial glucose 6-phosphate translocator leads to increased starch and tuber yield (Zhang et al., 2008).

A second strategy to influence ATP supply occurred through manipulation of a plastidial isoform of adenylate kinase, an enzyme that interconverts ATP with ADP and adenosine monophosphate (AMP) (Figure 1B). Repression of this enzyme led to increased ADP-glucose, tuber starch content, and yield (Regierer et al., 2002), presumably due to an increase in the plastidial ATP pool. Indeed, manipulation of nucleotide metabolism generally may be a profitable way to increase starch contents. In addition to the adenylate kinase study described above, repression of UMP synthase has been demonstrated to lead in uridine nucleotides accompanied by an increase in both cell wall and starch (Geigenberger et al., 2005). This is most likely caused by increases of flux into both components through increased sucrose degradation.

The work performed in altering substrate supply for AGPase has generally been performed in dicotyledonous plants. A recent study (Cakir et al., 2016) has examined rice plants where an increase in extra-plastidial AGPase activity was combined with overexpression of the plastidial ADP-glucose transporter (Figure 1B). They found that, although they could identify increased plastidial ADP-glucose amounts, this did not lead to an increase in starch, indicating additional stromal barriers affecting this pathway. If these could be identified, then they could be manipulated to allow increased starch accumulation.

### Can Alterations in Other Starch Biosynthetic Enzymes Increase Yield?

Although most work has examined influencing AGPase as a method of increasing starch amounts, it may not be the only protein that can do this. The polymerizing enzymes SS and SBE (Figure 1B) are present as multiple isoforms, which often play differing roles in determining the structure of the amylopectin molecule. It has been reported that increased expression of SS encoding genes is associated with increased starch accumulation and grain weight caused by expression of a mutated ubiquitin receptor in maize (Xie et al., 2018), while overexpression of one SS isoform increased potato tuber starch content (Gámez-Arjona et al., 2011). The data of Gámez-Arjona et al. (2011) have, however, been questioned (Sonnewald and Kossmann, 2013) due to the lack of dry matter increase that accompanied the reported starch elevation. Nevertheless, these data indicate that manipulation of SS isoforms may be a profitable way in increasing starch content.

There are fewer reports of SBE overexpression; however, one study in potatoes demonstrated that this led to synthesis of starch with altered structure but did not report on an effect on yield (Brummell et al., 2015). Interestingly, manipulation of SBE activity in Arabidopsis leaves has revealed a potential method for improving growth. Replacement of endogenous Arabidopsis activities with two from maize led to plants with increased starch in their leaves and improved seed yield, most likely also due to increased night-time sugar levels (Liu et al., 2016).

### Are Starch Degradative Enzymes Useful for Yield Increases?

Plant biotechnologists have generally attempted to increase starch yield through altering activities of enzymes involved in its synthesis. Degradative enzymes have often been ignored as targets for improving yield as mutations in them are often associated with reduced plant growth (Stitt and Zeeman, 2012; Paparelli et al., 2013). Recent work, however, has demonstrated that repression of the starch phosphorylating GWD enzyme (Figure 1A) in wheat endosperm improved both growth and seed production. Unfortunately, although this was demonstrated convincingly in glasshouse trials (Ral et al., 2012; Bowerman et al., 2016), when the same lines were examined in the field a reduction in yield was observed (Whan et al., 2017). Although it may not be a valuable way to increase starch yield in storage organs, repressing genes involved in this process could improve forage and silage crops by improving their calorific value for animal feed (Weise et al., 2012; Ruckle et al., 2018).

Manipulating starch degradation (Figure 1A) may have a greater role in helping plants overcome abiotic stress (Thalmann and Santelia, 2017). One of the main metabolites produced during starch catabolism is maltose, and it has been demonstrated that this can help stabilize photosynthetic membranes (Kaplan and Guy, 2004). *dpe2* mutant plants, which accumulate maltose, demonstrate reduced freezing damage (Li et al., 2011); however, they also grow smaller than wild-type plants (Chia et al., 2004). One strategy to overcome this may be the use of stress inducible promoters to drive repression constructs that reduce *Dpe2* expression only at times when increased maltose would be advantageous. Simultaneously, β-amylases that produce maltose during starch degradation (Kaplan and Guy, 2004; Kaplan et al., 2006) could be upregulated to increase levels of this metabolite further.

### Control Mechanisms Affecting Starch Metabolism

Although alterations of individual enzymatic steps within the starch pathway can have a beneficial effect on yield, alterations in transcriptional control mechanisms allow the possibility to influence these in a beneficial manner through altering multiple steps simultaneously. A few transcriptional regulators affecting starch metabolism have been identified (Zhang et al., 2005; Fu and Xue, 2010; Guan et al., 2011; Wang et al., 2013; Gontarek et al., 2016; Xiao et al., 2017), and manipulation of one of these led to increased seed size and yield in rice (Fu and Xue, 2010). More recently, transcriptional analysis has led to the identification of genes putatively involved in regulating starch metabolic genes (see for example Van Harsselaar et al., 2017), but their roles have often not been studied in detail. Such functional analyses would help in identification of factors that could be used to improve yield.

Post-transcriptional regulation will also be influential in controlling starch metabolism. Many sugar sensing mechanisms involving 14-3-3- and SnRK proteins, trehalose metabolism, TOR kinases, and hexokinase are known to affect starch metabolism. Alterations in expression of some 14-3-3 and SnRK proteins can lead to improved starch accumulation or to the upregulation of enzymes involved in starch synthesis, in several species (Sehnke et al., 2001; McKibbin et al., 2006; Wang et al., 2016, 2018). Trehalose 6-phosphate (T6P) has been proposed to activate AGPase through a post-translational redox mechanism (Kolbe et al., 2005), while genetic manipulation of T6P amounts can increase starch in leaves through repression of starch degradation (Martins et al., 2013) as well as increase yield in maize (Nuccio et al., 2015). More recently, it has been demonstrated that application of plant permeable analogues of T6P increases endosperm starch content and yield in wheat. The reasons for this are not entirely clear as many transcriptional and metabolic changes were identified; however, application of the same analog to Arabidopsis increased AGPase activity which would provide a direct explanation for the increased starch (Griffiths et al., 2016).

Post-translational alteration of proteins involved in starch metabolism can involve protein phosphorylation (Kötting et al., 2010) or reduction/oxidation mechanisms (Glaring et al., 2012; Santelia et al., 2015). Little is known about protein phosphorylation influencing starch metabolism, although a recent paper has identified plastidially localized protein kinases and phosphatases that may interact with starch metabolic enzymes (White-Gloria et al., 2018), which is a first step in the study of this process. More is known about redox control as several genes involved in starch metabolism are known to be redox regulated (Fu et al., 1998; Ballicora et al., 2000; Tiessen et al., 2002; Sokolov et al., 2006; Valerio et al., 2011; Glaring et al., 2012; Seung et al., 2013; Shaik et al., 2014), and expression of thioredoxin-f increased starch amounts in tobacco leaves (Sanz-Barrio et al., 2013), although it is not clear which enzymes were affected. The best characterization of the role of redox on starch metabolizing enzymes *in vivo* has been the examination of AGPase and GWD, where redox insensitive proteins have been expressed in plants (Haedrich et al., 2012; Skeffington et al., 2014). Redox insensitive AGPase led to increased leaf starch, although this was dependent on day length (Haedrich et al., 2012). On the other hand, constitutive expression of a redox insensitive GWD had little effect on leaf starch degradation (Skeffington et al., 2014), which agrees with the observation that reducing conditions (which would be expected to be present in chloroplasts during the day) activate the wild-type enzyme (Mikkelsen et al., 2005).

### Future Prospects

Although much has been achieved over the past decades in the manipulation of starch metabolism, there are still improvements that can be made. Detailed analysis of both transcriptional and post-translational control mechanisms will help fine tune current attempts at manipulating the pathway. Perhaps most importantly is the rational integration of metabolic engineering simultaneously in leaves and storage organs. One attempt to accomplish this involved reducing starch synthesis in potato leaves in order to increase soluble sugar export, while simultaneously increasing substrate supply to starch synthesis in tubers through overexpression of two plastidial transport proteins (Jonik et al., 2012). This approach led to an increase over and above the amounts found when either leaf or tuber metabolism was altered alone, doubling starch yield. We believe that such integrated approaches will lead to the greatest benefit for crop improvement.

## Author Contributions

JL wrote the first draft and edited the manuscript alongside JK.

### Conflict of Interest Statement

The authors declare that the research was conducted in the absence of any commercial or financial relationships that could be construed as a potential conflict of interest.
